# Inhibitory Effects of *Porphyra tenera* Extract on Oxidation and Inflammatory Responses

**DOI:** 10.1155/2021/6650037

**Published:** 2021-04-01

**Authors:** Chul Won Lee, Yong-Tae Ahn, Rongjie Zhao, Youn Sook Kim, Sang Mi Park, Dae Hwa Jung, Jae Kwang Kim, Hyung Woo Kim, Sang Chan Kim, Won G. An

**Affiliations:** ^1^Research Institute for Korean Medicine, Pusan National University, Yangsan 50612, Republic of Korea; ^2^Department of Psychopharmacology, Qiqihar Medical University, Qiqihar 161006, China; ^3^School of Medicine, Pusan National University, Yangsan 50612, Republic of Korea; ^4^College of Korean Medicine, Daegu Haany University, Gyeongsan 38610, Republic of Korea; ^5^Korean Medicine-Application Center, Korea Institute of Oriental Medicine, Daegu 41062, Republic of Korea; ^6^Division of Pharmacology, School of Korean Medicine, Pusan National University, Yangsan 50612, Republic of Korea

## Abstract

*Porphyra tenera* (laver) has long been a popular and traditional seaweed food in Korea, Japan, and China. Historically, it was known as a marine medicinal herb to treat hemorrhoids and cholera morbus in *Donguibogam*. We investigated the effects of *P. tenera* extract (PTE) for its antioxidant and anti-inflammatory activities. These activities were measured using assays for 2,2-diphenyl-1-picrylhydrazyl (DPPH) and nitric oxide (NO) radical scavenging and its superoxide dismutase- (SOD-) like activity, and through the inhibitory production of inflammatory mediators (prostaglandin E_2_ (PGE_2_), NO, tumor necrosis factor alpha (TNF-*α*), and interleukin-6 (IL-6)) in lipopolysaccharide- (LPS-) stimulated Raw 264.7 cells. The antioxidant assay results showed that PTE displayed DPPH radical scavenging activity (46.44%), NO radical scavenging activity (67.14%), and SOD-like activity (80.29%) at a concentration of 5 mg/mL. In the anti-inflammatory assays, treatment with PTE (1 mg/mL) significantly inhibited expression levels of LPS-induced COX-2 and iNOS, as well as the production of PGE_2_, NO, TNF-*α*, and IL-6. These results show that PTE has antioxidant and anti-inflammatory properties and provide scientific evidence to explain the antioxidative and anti-inflammatory properties of PTE.

## 1. Introduction

Inflammation is a host response to pathogen attack and is characterized by redness, heat, pain, and swelling. Over the long term, this response can lead to tissue damage and the pathogenesis of diverse disorders, such as atherosclerosis, asthma, and arthritis [1, 2]. Several inflammatory mediators are involved during an inflammatory response. Among them, COX-2, iNOS, and cytokines like IL-6 and TNF-*α* play significant roles and are considered as significant anti-inflammatory targets [3]. Moreover, when Raw 264.7 cells are activated, they produce reactive oxygen species (ROS), which cause oxidative stress. Oxidative stress is an inflammatory mediator that induces the release of nitric oxide (NO) and inflammatory cytokines [4]. NO is a radical made from L-arginine via NO synthase. NO contributes to the degeneration of inflammatory disorders, suppresses mitochondrial enzymes, and activates cyclooxygenases (COXs) to produce prostaglandins. In particular, the COX-2 enzyme is involved in the production of prostaglandin E_2_ (PGE_2_) [5, 6]. TNF-*α* is also a crucial mediator in the inflammatory response that leads to innate immune responses via the release of other inflammatory cytokines [7]. Conversely, IL-6 is produced by macrophages and is an important inflammatory cytokine in the acute phase response [8]. Accordingly, an inhibitor of NO, COX-2, ROS, and inflammatory cytokines is a crucial target for the treatment and prevention of inflammatory diseases.

*Porphyra tenera* (laver), a type of red algae (phylum: Rhodophyta, class: Bangiophyceae, order: Bangiales), has long been a popular and traditional seaweed food in Korea, Japan, and China [9]. *P. tenera* is rich in protein, carbohydrates, minerals, and vitamins and low in calories. Unlike other sea algae, *P. tenera* contains many free sugars, such as the major carbohydrates isofloridoside and floridoside. It also contains dietary fiber, including hemicellulose, which is a cell wall component and an insoluble polysaccharide [10, 11]. Moreover, *P. tenera* contains diverse inorganic and organic substances, including tocopherols, carotenoids, and polyphenols [12]. Importantly, *P. tenera* reportedly functions as a marine medicinal herb for the treatment of hemorrhoids and cholera morbus in *Donguibogam* [13]. It is reported that *P. tenera* functions as an antioxidant [14], has anti-inflammatory activities [15], and exerts antimutagenic effects [16]. Furthermore, Song et al. [17] reported that *P. tenera* extract (PTE) activates the immune response in mouse Raw 264.7 cells via NF-*κ*B signaling. However, the molecular mechanisms underlying the antioxidative and anti-inflammatory activities of PTE remain unknown. In the present study, we examined the antioxidant and anti-inflammatory profile of PTE. Our data provide a basis for understanding the mechanisms underlying the inhibitory effects of PTE on oxidation and inflammatory responses.

## 2. Materials and Methods

### 2.1. Chemicals and Reagents

Two reference standards, chlorogenic acid and palmitic acid, were purchased from Sigma Aldrich Chemical Co. (St. Louis, MO, USA). The purity of these reference standards was greater than 98%. Ultra-high-performance liquid chromatography- (UPLC-) grade solution, acetonitrile, methanol, and other reagents were purchased from J. T. Baker Chemical Company (Phillipsburg, NJ, USA). Anti-COX-2 and peroxidase-conjugated secondary antibodies were obtained from Santa Cruz Biotechnology Inc. (Santa Cruz, CA, USA). In addition, anti-*β*-actin and anti-iNOS antibodies were obtained from Calbiochem (San Diego, USA.). The enzyme-linked immunoassay kit of PGE_2_ was obtained from R&D Systems (Minneapolis, USA), and the TNF-*α* and IL-6 enzyme immunosorbent assay kits were purchased from Pierce Endogen (Rockford, USA). MTT, LPS, sulfanilamide, L-N^6^-(1-Iminoethyl)lysine (L-NIL), NS-398, and all other chemicals were purchased from the Sigma Chemical Co. (St. Louis, MO, USA).

### 2.2. Preparation of PTE

*P. tenera* was purchased from Jindoherb Co. (Jindo, Jeollanam-do, Korea). The voucher specimens (*Porphyra tenera,* PNU10-150) have been deposited into the Herbarium of Ducom in the Korean Medicine, Pusan National University, South Korea. *P. tenera* (50 g) was extracted with 3 liters of boiling purified water for 3 h long and filtered via a filter paper (Advantec No. 2 Filter Paper; Advantec Toyo Kaisha, Ltd., Tokyo, Japan). The filtrate was then lyophilized in a freeze drier (Ilshin, Seoul, Korea). The yield of lyophilized PTE was 2.32%. The lyophilized PTE powder was dissolved in purified water and filtered via a 0.22 *μ*m filter (Nalgene, USA) prior to use.

### 2.3. Profiling the Chemical Contents of PTE by UPLC

#### 2.3.1. Chromatography Conditions

We applied an UPLC (Waters Corp., Milford, USA), supplied with a Waters pump ACQUITY™ UltraPerformance LC system (Waters Corp.) and a Waters ACQUITY™ photodiode array (PDA) detector, for the analyses. The Empower Chromatography Data System (Waters Corp.) was used to record the output signal from the detector and a Waters ACQUITY™ BEH C_18_ column (1.7 *μ*m, 2.1 × 100) was used for separation of the products. The mobile stage was constituted of acetonitrile and water with a gradient system (0.4 mL per min). The volume for injection was 2 *μ*L. The UV wavelength for detection was set up at 280 nm. The temperature for the column was set up at 22–25°C.

#### 2.3.2. Preparation of the Standard Solutions and Samples

Standard stock solutions of the marker components, chlorogenic acid, and palmitic acid, were prepared by dissolving them at a concentration of 1 mg/mL in 10 mL methanol. Working solutions were produced by diluting the stock solution of standard with methanol. The standard stock solutions and working solutions were stored at 4°C. For the sample preparation, the PTE was dissolved in methanol (10 mg/mL). Before UPLC, the sample was filtered via a 0.22 *μ*m filter.

### 2.4. Antioxidant Assays

#### 2.4.1. DPPH Radical Scavenging Activity Assay

Electron donating ability was evaluated using 1,1-diphenyl-2-picrylhydrazyl (DPPH) by the method of Blois [18]. Briefly, 100 *μ*L of DPPH solution (0.4 mM in ethanol) was added to 100 *μ*L of PTE (dissolved in ethanol) at concentrations of 0.1–5 mg/mL. Ethanol was used as the control for the experiments. The mixture was incubated for 15 min at 22–25°C. The optical density was measured at 517 nm by a microplate reader (Tecan Group Ltd., Männedorf, Switzerland). Ascorbic acid was applied as a positive control. The capacity to scavenge the DPPH radical was calculated by the subsequent formula:

(1) $$\begin{array}{c} \text{DPPH radical scavenging activity}\left(\%\right)=\frac{\left({\text{absorbance}}_{\text{control}}-{\text{absorbance}}_{\text{treatment}}\right)}{{\text{absorbance}}_{\text{control}}}\times 100, \end{array}$$

where absorbance_control_ and absorbance_treatment_ are the absorbance of the control and the treatment, respectively.

#### 2.4.2. NO Radical Scavenging Activity Assay

NO radical scavenging activity was measured according to Kato et al. [19]. Briefly, 40 *μ*L of each sample concentration was added to 20 *μ*L of 1 mM NaNO_2_ solution, followed by the addition of 140 *μ*L of 0.1 N HCl (pH 1.2). The mixture was allowed to react at 37°C for 1 h. Next, 40 *μ*L of the reaction mixture was added to 200 *μ*L of 2% acetic acid, followed by mixing with 16 *μ*L of Griess reagent. After incubating at 22–25°C for 15 min, the optical density was measured at 520 nm by a microplate reader (Tecan Group Ltd.). Ascorbic acid was applied as a positive control. The NO radical scavenging activity was calculated as (%) = (1 − (*A* − *B*)/*C*) × 100, where *A* is the optical density of the sample without Griess reagent, *B* is the absorbance of the sample with Griess reagent, and *C* is the absorbance of the control.

#### 2.4.3. SOD-like Activity Assay

The SOD-like activity was evaluated by determining the amount of pyrogallol needed to catalyze the conversion to H_2_O_2_, based on Marklund and Marklund [20]. The reaction mixture contained 20 *μ*L of the sample (10 mg/mL) and 300 *μ*L of 50 mM Tris-HCl buffer (pH 8.5), which were mixed with 10 mM EDTA and 20 *μ*L of 7.2 mM pyrogallol. The mixture was incubated at room temperature for 10 min, and then the reaction was blocked by adding 10 *μ*L 1 N HCl. The optical density was measured at 420 nm by a microplate reader (Tecan Group Ltd.). Ascorbic acid was applied as a positive control. The SOD-like activity was calculated with the following formula:

(2) $$\begin{array}{c} \text{SOD}-\text{like activity}\left(\%\right)=\frac{\left({\text{absorbance}}_{\text{control}}-{\text{absorbance}}_{\text{treatment}}\right)}{{\text{absorbance}}_{\text{control}}}\times 100, \end{array}$$

where absorbance_control_ and absorbance_treatment_ are the absorbance of the control and the treatment, respectively.

### 2.5. Cell Culture

Raw 264.7 cells (ATCC, Manassas, USA) were maintained in DMEM (Hyclone; Thermo Fisher Scientific, Waltham, USA) supplemented with 10% heat-inactivated fetal bovine serum (Sigma Aldrich Chemical Co.), 100 *μ*g/mL of streptomycin, and 100 U/mL of penicillin (Gibco-BRL, Grand Island, USA) in a 5% CO_2_ incubator at 37°C.

### 2.6. MTT Assay for Cell Viability

We followed the methods of Park et al. [21]. Briefly, to determine the cytotoxic concentration of PTE, Raw 264, 7 cells were planted in 96 wells (5 × 10^4^ cells per well). Cells were serum-starved for 16 h, treated with various concentrations of PTE for 1 h, induced by 1 *μ*g/mL of LPS, and then the cells were incubated for 20 h at 37°C in an incubator with 5% CO_2_. Following incubation, cells were stained with MTT at the concentration of 0.5 mg/mL for 4 h, and then the media were eliminated and the formazan produced was dissolved by adding 200 *μ*L DMSO. Optical density was measured at 570 nm by an ELISA plate reader (Tecan Group Ltd.). Cell viability was described relative to the untreated control cells, where viability (% control) = 100 × (optical density of treated sample)/(optical density of control).

### 2.7. PGE_2_ and Cytokines (TNF-*α* and IL-6) Assays

Cells (Raw 264.7 macrophage, 5 × 10^5^ cells/mL) were incubated for 16 h. The cells were then treated with various concentrations of PTE or with a positive control for the production of PGE_2_ (NS-398) for 1 h, followed by stimulation with 1 *μ*g/mL of LPS. At 20 h after LPS stimulation the culture supernatants were collected and ELISA was performed according to the manufacturer's protocol to quantify the amounts of PGE_2_, TNF-*α*, and IL-6 (PGE_2_, R&D Systems; TNF-*α* and IL-6, Pierce Endogen).

### 2.8. Measurement of NO Production

The Raw 264.7 cells (5 × 10^5^ cells/mL) were incubated for 16 h, after which the cells were pretreated with various concentrations of PTE or with a positive control (L-NIL) for 1 h and induced by LPS (1 *μ*g/mL). Next, the cells were incubated for 20 h at 37°C in a 5% CO_2_ incubator, after which the culture supernatants were collected. NO was measured by adding 100 *μ*L of Griess reagent (0.1% *N*-[1-naphthy]-ethylenediamine dihydrochloride and 1% sulfanilamide in 5% phosphoric acid; Roche, Switzerland) to the culture supernatant (100 *μ*L) for 15 min at 22–25°C in the dark. Optical density was determined at 540 nm by an ELISA plate reader (Tecan Group Ltd.). A standard curve was generated similar to that of NaNO_2_.

### 2.9. Western Blot Analysis

Control and PTE-treated Raw 264.7 cells were harvested by centrifugation and washed twice with phosphate-buffered saline (PBS). Washed pellets of cells were resuspended in lysis buffer for extraction (0.5 mM dithiothreitol, 5 mM EDTA, 250 mM NaCl, 5 mM NaF, 0.1% Nonidet P-40, 1 mM phenylmethylsulfonyl fluoride, 0.5 mM sodium orthovanadate, and 50 mM HEPES (pH 7.0)) containing 5 *μ*g/mL each of aprotinin and leupeptin and incubated at 4°C for 20 min. Microcentrifugation was performed to remove the cell debris, followed by rapid freezing of the supernatant. Bio-Rad protein assay reagent was used to measure the protein concentrations by the manufacturer's instructions. Then, the cellular proteins (30 *μ*g) from cell extracts were separated on 8% sodium dodecyl sulfate–polyacrylamide gels, followed by electroblotting onto nitrocellulose membranes. The membranes were incubated overnight, shaking with 5% skim milk at 4°C and subsequently with primary antibody (2 h). The blots were then washed five times with tween 20/tris-buffered saline (TTBS), incubated for 1 h, shaking with a 1 : 1000 dilution of horseradish peroxidase-conjugated secondary antibody at 22–25°C, and then rewashed three times with TTBS. ECL™ Western Blot Reagents for Detection (Amersham Biosciences, USA) were used to develop the blots.

### 2.10. Statistical Analysis

All data were recorded as means ± standard deviation (SD). The data were estimated by one-way analysis of variance (ANOVA) tests, Dunnett's tests, and independent *t*-tests. SPSS for Windows (release 25.0 K, SPSS Inc., USA) was used for all statistical analyses. Differences were considered significant at *p* < 0.05.

## 3. Results

### 3.1. PTE Analysis

**Figure 1:**
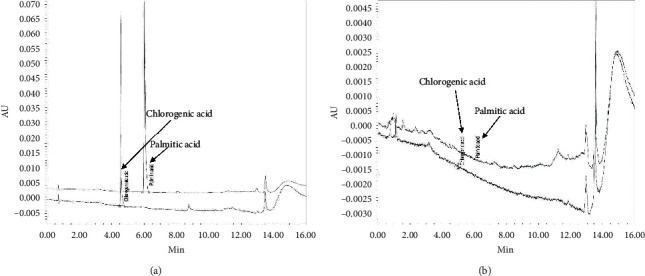
UPLC chromatogram of two marker compounds in PTE. UPLC chromatogram of standard compounds (a). UPLC chromatogram of two marker compounds in PTE (b). The chromatograms were obtained at 280 nm.

UPLC was used to identify two PTE markers: chlorogenic acid and palmitic acid. Their contents were calculated from the standards calibration curve (Figure 1 and Table 1). Validation of the method proved its stability and reliability. UPLC resulted in the successive separation of the two marker components in PTE.

### 3.2. Antioxidant Assay

#### 3.2.1. DPPH Scavenging Activity

**Figure 2:**
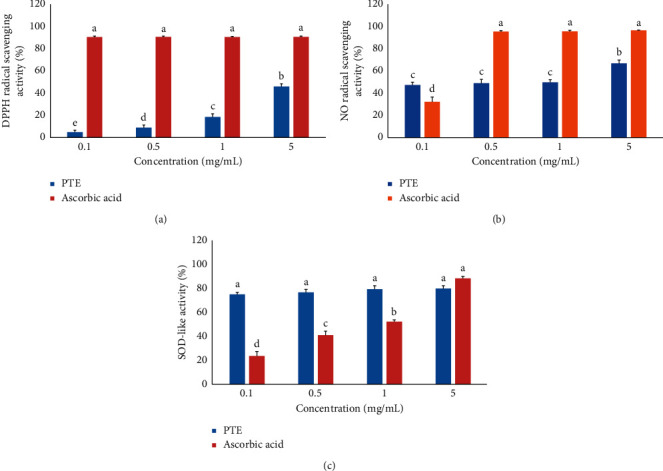
Antioxidant activities of PTE. Free radical scavenging activities of PTE were measured based on the detection of the DPPH (a) and NO (b) radical scavenging activities. SOD-like activity (c) was evaluated using the pyrogallol method. Values are expressed as the means ± SD from three experiments. Different letters indicate significant differences among the groups (*p* < 0.05).

Antioxidative activity of PTE was determined using DPPH radicals to ascertain the free radical scavenging activity (0.1–5 mg/mL). The DPPH radical scavenging activity increased significantly as the concentration of PTE increased (*p* < 0.05, Figure 2(a)). PTE displayed scavenging activities of 18.71% and 46.44% at concentrations of 1 and 5 mg/mL, respectively. Although the DPPH radical scavenging activity of PTE at 5 mg/mL reached 46%, that of the reference compound, ascorbic acid displayed more scavenging activity (∼91%) than PTE at concentrations of 0.1–5 mg/mL.

#### 3.2.2. NO Radical Scavenging Activity

Figure 2(b) presents the NO radical scavenging potential of PTE alongside that of ascorbic acid as the positive control. PTE exhibited scavenging activities of 50.01% and 67.14% at concentrations of 1 and 5 mg/mL (*p* < 0.05), respectively, whereas ascorbic acid exhibited scavenging activities of 96.09% and 97.06% at concentrations of 1 and 5 mg/mL, respectively.

#### 3.2.3. SOD-like Activity

PTE at concentrations of 0.1–5 mg/mL exhibited a slight dose-dependent effect in terms of SOD-like activity (Figure 2(c)). PTE exhibited SOD-like activities of 79.84% and 80.29% at 1 and 5 mg/mL, respectively. The reference compound ascorbic acid exhibited SOD-like activities of 52.53% and 88.85% at 1 and 5 mg/mL, respectively (*p* < 0.05).

### 3.3. Inhibitory Effects of PTE on LPS-Induced Production of NO and PGE_2_

**Figure 3:**
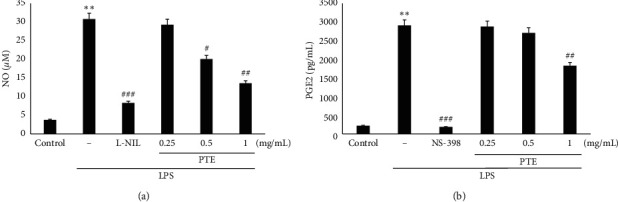
Inhibitory effects of PTE on LPS-induced production of NO (a) and PGE_2_ (b) in Raw 264.7 cells. Cells (5 × 10^5^ cells/mL) were treated with 0.25, 0.5, or 1 mg/mL PTE for 1 h followed by continuous incubation with 1 *μ*g/mL LPS for the next 20 h L-N^6^-(1-iminoethyl)lysine (L-NIL) and NS-398 (10 *μ*M each) were used as positive controls, respectively. Concentrations of NO and PGE_2_ in the culture medium were monitored as described in methods. Data show the means ± SD from three experiments. *∗∗p* < 0.01 compared with the control; ###*p* < 0.001, ##*p* < 0.01, and #*p* < 0.05 compared with LPS alone.

Different concentrations of PTE (0.25–1 mg/mL) were used to evaluate the inhibitory effects of PTE on LPS-stimulated production of NO and PGE_2_ in Raw 264.7 cells. Compared to the control, treatment with LPS resulted in significantly increased NO production. However, treatment with L-NIL (10 *μ*M), a positive control, significantly reduced the production (*p* < 0.001) of LPS-induced NO. In addition, treatment with PTE resulted in significantly reduced production of LPS-induced NO at concentrations of 0.5 and 1 mg/mL (#*p* < 0.05 for 0.5 mg/mL, ##*p* < 0.01 for 1 mg/mL; Figure 3(a)). Furthermore, we observed the same inhibitory effects of PTE on LPS-induced PGE_2_ production (Figure 3(b)). Compared to the control, treatment with LPS resulted in significantly increased PGE_2_ production. However, treatment with NS-398 (10 *μ*M), a positive control, significantly reduced the production (*p* < 0.001) of LPS-induced PGE_2_. Moreover, treatment with PTE resulted in significantly reduced LPS-induced PGE_2_ production at a concentration of 1 mg/mL (##*p* < 0.01 for 1 mg/mL; Figure 3(b)). Thus, PTE exhibited an inhibitory effect on the induction of NO and PGE_2_ in Raw 264.7 cells.

### 3.4. Cell Viability

**Figure 4:**
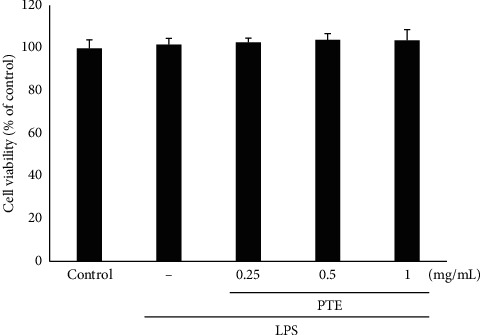
Viability of cells exposed to PTE based on the MTT assay. Data show the means ± SD from three separate experiments.

We used the MTT assay to test for possible cytotoxic effects of PTE in Raw 264.7 cells. There were no changes in cell viability after exposure to 0.25, 0.5, and 1 mg/mL PTE (Figure 4), indicating that PTE displayed no cell toxicity.

### 3.5. Inhibitory Effects of PTE on LPS-Stimulated Expression of COX-2 and iNOS

**Figure 5:**
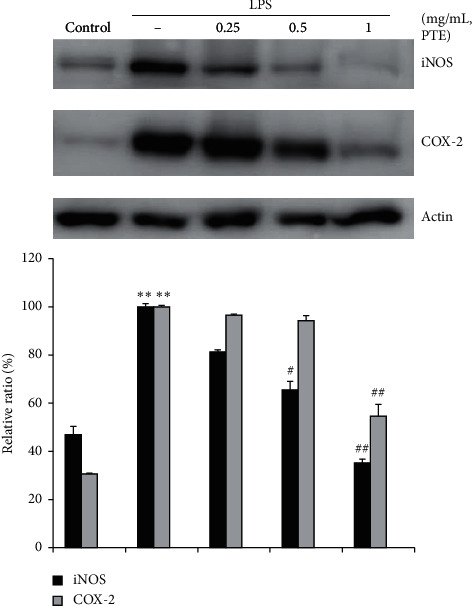
Inhibitory effects of PTE on LPS-stimulated expression of COX-2 and iNOS. Raw 264.7 macrophage cells (5 × 10^5^ cells/mL) were treated with PTE (0.25–1 mg/mL) for 1 h followed by continuous incubation with 1 *μ*g/mL LPS for the next 20 h. The cells of control were incubated with the vehicle only. Western blot analysis was carried out to determine the COX-2 and iNOS protein levels. *β*-Actin was used as a control. The blots are representative results of three blots. COX-2 and iNOS versus *β*-actin were determined through densitometry. Data show the means ± SD from three experiments. *∗∗p* < 0.01 between the control and LPS-treated cells; #*p* < 0.05 and ##*p* < 0.01 between the LPS-treated cells with, or without PTE.

Western blot analysis was carried out to determine whether the effects of PTE against PGE_2_ and NO production were related to modulation of COX-2 and iNOS. There were marked increases in the levels of COX-2 and iNOS proteins in response to LPS (Figure 5). PTE (1 mg/mL) showed significant suppression of LPS-stimulated COX-2 protein levels (##*p* < 0.01 for 1 mg/mL). In addition, treatment with PTE (0.5 and 1 mg/mL) resulted in significant inhibition of LPS-stimulated iNOS protein levels (#*p* < 0.05 for 0.5 mg/mL, ##*p* < 0.01 for 1 mg/mL). These data confirmed the inhibitory effects of PTE on the production of PGE_2_ and NO in LPS-induced Raw 264.7 macrophage cells.

### 3.6. Inhibitory Effects of PTE on LPS-Stimulated TNF-*α* and IL-6 Production

**Figure 6:**
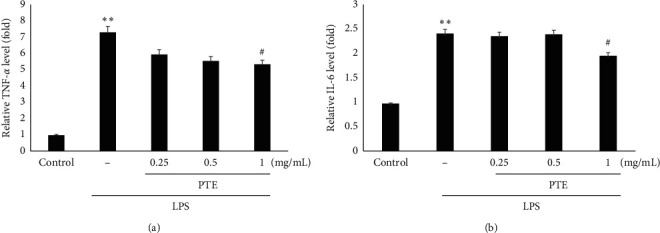
Inhibitory effects of PTE on LPS-stimulated TNF-*α* (a) and IL-6 (b) production in Raw 264.7 macrophage cells. Cells (5 × 10^5^ cells/mL) were treated with PTE (0.25–1 mg/mL) for 1 h followed by continuous incubation with 1 *μ*g/mL LPS for the next 20 h TNF-*α* and IL-6 concentrations in the culture supernatants were recorded as described in Section 2. Data show the means ± SD from three experiments. *∗∗p* < 0.01 compared to the control; #*p* < 0.05 compared with LPS alone.

We performed enzyme immunoassays to evaluate the effects of PTE on the LPS-inducible production of TNF-*α* and IL-6. Compared to the control, LPS treatment showed a significant increase of TNF-*α* and IL-6 in the culture supernatants of Raw 264.7 macrophage cells (*p* < 0.01). However, treatment with 1 mg/mL PTE showed significant inhibition in LPS-induced TNF-*α* and IL-6 production (Figures 6(a) and 6(b)). These findings indicate that PTE might inhibit the expression of the specific genes involved in the inflammation response, such as TNF-*α* and IL-6.

## 4. Discussion

*P. tenera* has long been a popular and traditional seaweed food in Korea, Japan, and China [9]. It is also reported to have antioxidative [14] and anti-inflammatory effects [15]. However, there is little scientific evidence to demonstrate the effects of PTE. Consequently, we examined the molecular mechanisms underlying the antioxidative and anti-inflammatory effects of PTE.

ROS consist of hydroxyl radicals, peroxynitrite, singlet oxygen, peroxyl radicals, and superoxide, which cause oxidative stress, leading to cellular damage [22]. Identifying the free radical-quenching abilities and antioxidant activities of antioxidant compounds sourced from plants is essential [23]. In the present study, we used DPPH [18] and NO [19] radical scavenging, as well as SOD-like activity [20], assays to estimate the antioxidant activity of PTE. DPPH is commonly used in antioxidant assays [24]; in this study, the DPPH radical scavenging activity was significantly increased as the concentration of PTE increased. PTE had a DPPH radical scavenging activity of 46.44% at a concentration of 5 mg/mL; however, the reference compound, ascorbic acid, showed higher scavenging activity (91.53%) compared to PTE at a concentration of 5 mg/mL. NO is unstable in the aerobic state; it reacts with O_2_ to make stable nitrite and nitrate products via the N_3_O_4_, N_2_O_4_, and NO_2_ intermediates [25]. Our results indicated that 5 mg/mL PTE exhibited a NO radical scavenging activity of 67.14%, whereas 5 mg/mL ascorbic acid showed a scavenging activity of 97.06%. SOD is a significant antioxidant enzyme catalyzing the conversion of the superoxide radical into H_2_O_2_ and O_2_ [26]. We measured the SOD-like activity of PTE at different concentrations (0.1–5 mg/mL) and observed slight dose-dependent responses. At 5 mg/mL, PTE showed a SOD-like activity of 80.29%, whereas 5 mg/mL ascorbic acid exhibited a SOD-like activity of 88.85% (*p* < 0.05). Collectively, these results suggest that the effects of PTE on the DPPH, NO radical scavenging, and SOD-like activities might have important implications for strategies used to manage pathological stress.

In wounds, inflammation is associated with microbiological toxins or chemicals [1]. LPS stimulates the production of the NO, PGE_2_, and cytokines by initiating the NF-*κ*B transcription factor in Raw 264.7 cells [27–29]. The inflammatory cytokines TNF-*α* and IL-6 are involved in various immunological interactions and reactions with diverse target cells [6, 7, 30]. In this study, we evaluated the inhibitory effects of PTE on LPS-stimulated TNF-*α* and IL-6 by ELISA, which revealed that PTE (1 mg/mL) significantly inhibited LPS-induced levels of cytokines (TNF-*α* and IL-6). Thus, the inhibitory effects of PTE on inflammatory cytokines could form the basis for treatments of pathological inflammation. Moreover, iNOS causes damage to cells via NO production in macrophages stimulated by LPS [31]. In this study, PTE significantly suppressed LPS-induced iNOS protein expression and NO production.

Furthermore, treatment with L-NIL (10 *μ*M), a positive control, significantly reduced the production of LPS-induced NO. In particular, the NO production value for treatment with PTE (1 mg/mL) was somewhat higher than that of the L-NIL. This finding indicates that PTE, similar to the L-NIL, can inhibit NO production. In addition, these results suggest that the preventive effects of PTE on the inflammatory response are due, in part, to its suppression of iNOS expression and NO production. Furthermore, PGE_2_, made via COX-2 from arachidonic acid, plays crucial regulatory roles in the inflammatory responses and in brain injuries [32]. PGE_2_ is released from blood vessel walls in response to inflammation or infection to induce fever [33]. Therefore, inhibition of PGE_2_ is useful to help identify beneficial plant extracts that have anti-inflammatory properties [34, 35]. Our data showed that PTE resulted in significant inhibition of PGE_2_ and the COX-2 protein induced by LPS. Moreover, treatment with NS-398 (10 *μ*M), a positive control, significantly reduced the production of LPS-induced PGE_2_. In particular, the PGE_2_ production value for treatment with PTE (1 mg/mL) was higher than that of the NS-398. This result shows that PTE can weakly inhibit PGE_2_ production compared to the NS-398. Therefore, these findings suggest that PTE might have preventive and therapeutic effects in the treatment of pathogenic pain, heat, and inflammation.

PTE is an aqueous extract of the red algae *P. tenera*. Zhang et al. [36] reported that the sulfated galactan fraction isolated from the red seaweed *Porphyra haitanensis* had significant *in vivo* antioxidant activity in aging mice. In addition, Senevirathne et al. [37] showed that enzymatic extracts from *P. tenera* effectively inhibited LPS-stimulated production of NO in Raw 264.7 macrophage cells. Furthermore, the red algae *Porphyra yezoensis* reportedly showed high antitumor activity against Ehrlich carcinoma [38]. According to Jung et al. [39], high-performance liquid chromatography showed that PTE contained *Porphyra*-334. However, our chromatographic results revealed that the main markers of PTE were chlorogenic acid and palmitic acid (Table 1 and Figure 1). Chlorogenic acid, an abundant polyphenol compound, possesses multiple biological activities, including anti-inflammation, immunomodulation, antivirus, and cardiovascular protection [40–42]. In addition, chlorogenic acid reportedly has a protective effect on myocardial infarction via its antioxidant activity and reducing the inflammatory response [43]. Meanwhile, palmitic acid is a saturated fatty acid discovered in microorganisms, plants, and animals. It is naturally produced by a wide range of plants and organisms, but typically at low levels [44]. From the findings from this study, we suggest that the antioxidative and anti-inflammatory activities of PTE are likely due to chlorogenic acid.

## 5. Conclusion

We showed that PTE has antioxidative and anti-inflammatory properties using DPPH, NO radical scavenging, and SOD-like activity assays and that it acts through the inhibitory actions of inflammatory mediators (NO, PGE_2_, TNF-*α*, and IL-6) in LPS-induced Raw 264.7 macrophage cells. Our results provide scientific evidence that explains the antioxidant and anti-inflammatory properties of PTE.

## Figures and Tables

**Table 1:** Contents of two marker compounds in PTE by UPLC (*n* = 3).

Compound	Content (*μ*g/g)
Chlorogenic acid	3.016 ± 0.061
Palmitic acid	9.212 ± 0.002

## Data Availability

The data justifying the conclusions of this study are all statistically analyzed and presented in the Results section and are also available from the corresponding authors.
